# The genome sequence of *Alnus cordata* (Loisel. Duby, 1828) (Fagales: Betulaceae)

**DOI:** 10.12688/wellcomeopenres.27529.1

**Published:** 2026-09-16

**Authors:** Markus Ruhsam, Peter M. Hollingsworth, Royal Botanic Garden Edinburgh Genome Acquisition Lab, Plant Genome Sizing Collective, Darwin Tree of Life Barcoding collective, Wellcome Sanger Institute Tree of Life Management, Samples and Laboratory team, Wellcome Sanger Institute Scientific Operations: Sequencing Operations, Wellcome Sanger Institute Tree of Life Core Informatics team, Tree of Life Core Informatics collective, Darwin Tree of Life Consortium

**Affiliations:** 1 Royal Botanic Garden Edinburgh, Edinburgh, Scotland, UK

**Keywords:** Alnus cordata, Italian Alder, genome sequence, chromosomal, Fagales

## Abstract

We present a genome assembly of *Alnus cordata* (Italian Alder; Streptophyta; Magnoliopsida; Fagales; Betulaceae). The assembly consists of two haplotypes with total lengths of 468.52 megabases and 468.51 megabases. Most of haplotype 1 (98.99%) is scaffolded into 14 chromosomal pseudomolecules. Haplotype 2 was assembled to scaffold level. The mitochondrial sequences have lengths of 521.58 and 155.53 kilobases and the plastid genome assembly has a length of 160.76 kilobases. This assembly was generated as part of the Darwin Tree of Life project, which produces reference genomes for eukaryotic species found in Britain and Ireland.

## Species taxonomy

Eukaryota; Viridiplantae; Streptophyta; Streptophytina; Embryophyta; Tracheophyta; Euphyllophyta; Spermatophyta; Magnoliopsida; Mesangiospermae; eudicotyledons; Gunneridae; Pentapetalae; rosids; fabids; Fagales; Betulaceae; *Alnus*; *Alnus cordata* (Loisel. Duby, 1828) (NCBI:txid109058).

## Background

*Alnus cordata* is a deciduous broadleaved tree endemic to the southern Apennine Mountains of Italy and north-eastern Corsica, where it occurs in fragmented natural populations ( Ducci & Tani, 2009; Pollegioni *et al.*, 2025; Villani *et al.*, 2021). Within its native range, the species inhabits mountainous environments and occurs from approximately 200 to 1,600 m elevation depending on rainfall and temperature ( Ducci & Tani, 2009). Italian alder is a light-demanding pioneer species that readily colonises forest gaps and disturbed sites, particularly within beech ( *Fagus sylvatica*) and chestnut ( *Castanea sativa*) stands ( Ducci & Tani, 2009). Unlike many other alder species, *A. cordata* is less dependent on riparian habitats and exhibits relatively high drought tolerance, although it commonly occurs in areas with annual precipitation exceeding 1,000 mm ( Caudullo & Mauri, 2016).

*Alnus cordata* is a monoecious and wind-pollinated species, producing separate male and female inflorescences on the same individual. Male inflorescences are pendulous catkins, while female inflorescences develop into woody cone-like structures that persist after seed release ( Ducci & Tani, 2009). The species reaches reproductive maturity after about 10 to 12 years under natural conditions ( Ducci & Tani, 2009). Recent genetic studies indicate that *Alnus cordata* populations exhibit relatively low genetic diversity, with diversity declining towards the southern latitudinal margins of the Italian peninsula ( Pollegioni *et al.*, 2025). Evidence of introgression with *Alnus glutinosa* has also been detected in some populations, potentially contributing to local patterns of genetic variation ( Pollegioni *et al.*, 2025; Villani *et al.*, 2021); however hybrid individuals have limited capacity for backcrossing to the parental species, reducing the risk of genetic pollution ( Villani *et al.*, 2021).

The chemistry of *A. cordata* is characterised by a diverse array of phenolic compounds, particularly in stem bark tissues. Phytochemical analyses of methanolic stem bark extracts revealed a high content of hydroxycinnamic acids, flavanones and diarylheptanoids, and the extracts exhibited strong antioxidant and free-radical scavenging activity ( Smeriglio *et al.*, 2019).

Like other members of the genus, *A. cordata* forms symbiotic associations with nitrogen-fixing actinomycetes of the genus *Frankia*, thereby improving soil fertility through biological nitrogen fixation ( Caudullo & Mauri, 2016; Ducci & Tani, 2009). Italian alder may be susceptible to diseases caused by oomycetes of the *Phytophthora alni* complex, pathogens associated with alder decline across Europe ( Brasier *et al.*, 2004). Owing to its rapid growth, drought tolerance and soil-improving properties, *A. cordata* has become an important species in ecological restoration, land reclamation and urban forestry throughout Europe ( Caudullo & Mauri, 2016).

The chromosome number of *Alnus cordata* has been reported as 2 *n* = 28 and 2 *n* = 42 in the Chromosome Counts Database ( Rice & Mayrose, 2023). If the commonly reported basic chromosome number of *x* = 14 for this genus is assumed, these counts correspond to diploid and triploid cytotypes, respectively. In contrast, *A. glutinosa* is predominantly diploid (2 *n* = 28), but a recent cytotype study has also revealed mixed-ploidy populations including diploids, tetraploids, and occasionally triploids across parts of its European range ( Vít *et al.*, 2017).

We present a chromosome-level genome sequence for *A. cordata.* The assembly was produced using the Tree of Life pipeline from a specimen collected in North Berwick, East Lothian, Scotland ( Figure 1). It was generated as part of the Darwin Tree of Life Project, which aims to generate high-quality reference genomes for all named eukaryotic species in Britain and Ireland to support research, conservation, and the sustainable use of biodiversity ( Darwin Tree of Life Project Consortium, 2022).

**Figure 1.**
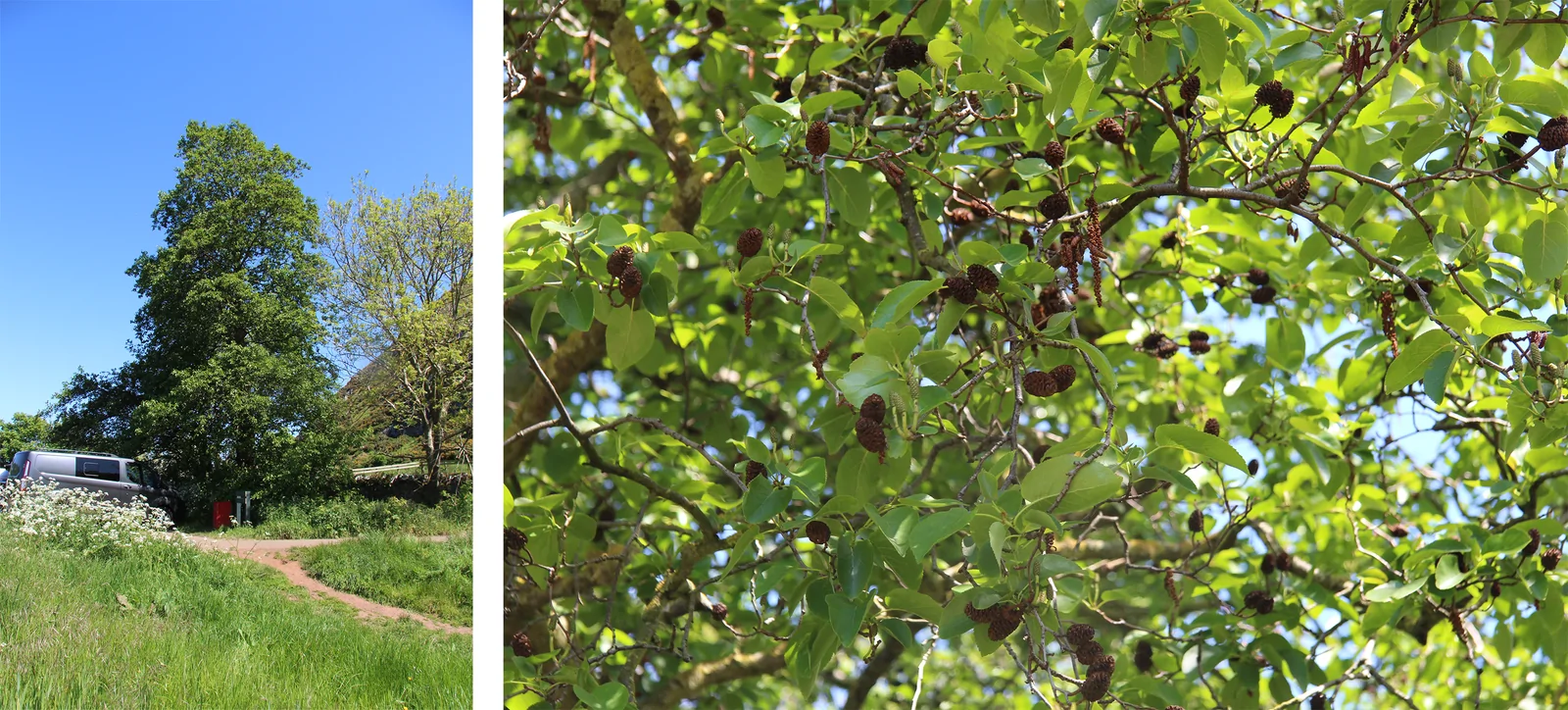
*Alnus cordata* (dhAlnCord1) from which samples were taken for genome sequencing.

## Methods

### Sample acquisition, flow cytometry and DNA barcoding

A specimen of *Alnus cordata* (specimen ID EDTOL05638, ToLID dhAlnCord1; Figure 1) was used for genome sequencing. It was collected from North Berwick, UK (latitude 56.0489, longitude −2.719) on 2023-05-30. The specimen was collected and identified by Markus Ruhsam (Royal Botanic Garden Edinburgh). The dhAlnCord1 specimen was also used for Hi-C and RNA sequencing. The herbarium voucher associated with the sequenced plant is E01152975 and is deposited in the herbarium of RBG Edinburgh (E) https://data.rbge.org.uk/herb/E01152975.

The genome size was estimated by flow cytometry following the ‘one-step’ method outlined in Pellicer *et al*. (2021) and using propidium iodide as the fluorochrome. Galbraith was used for isolation of nuclei ( Loureiro *et al.*, 2007), and the internal calibration standard was *Petroselinum crispum* ‘Champion Moss Curled’ with an assumed 1C-value of 2 200 Mb ( Obermayer *et al.*, 2002).

The initial identification was verified by an additional DNA barcoding process according to the framework developed by Twyford *et al*. (2024). Part of the plant specimen was preserved in silica gel desiccant ( Chase & Hills, 1991). DNA extracted from the dried plant was amplified by PCR for standard barcode markers, with the amplicons sequenced and compared to public sequence databases including GenBank and the Barcode of Life Database (BOLD) ( Ratnasingham & Hebert, 2007). Following whole genome sequence generation, the relevant DNA barcode region was also used alongside the initial barcoding data for sample tracking at the WSI ( Twyford *et al.*, 2024). The standard operating procedures for Darwin Tree of Life barcoding are available on protocols.io.

### Nucleic acid extraction

High molecular weight (HMW) DNA extraction protocols developed by the Tree of Life Core Laboratory at the Wellcome Sanger Institute (WSI) are available on protocols.io ( Howard *et al.*, 2025). Tissue from the dhAlnCord1 sample was weighed and triaged to select the appropriate extraction workflow. HMW DNA was prepared for one PacBio HiFi library: DTOL14377264. For this library, 68.0 mg of leaf tissue was used. The tissue was homogenised using Bead Beating. HMW DNA was extracted using the Plant MagAttract 48xrn v4 protocol. DNA was fragmented following the Sanger Tree of Life HMW DNA Fragmentation: Diagenode Megaruptor3 for LI PacBio protocol. Sheared DNA was purified following the Sanger Tree of Life Fragmented DNA clean up: Manual SPRI protocol.

The concentration of the sheared and purified DNA was assessed using a NanoDrop spectrophotometer and Qubit Fluorometer using the Qubit dsDNA High Sensitivity Assay kit. Fragment size distribution was evaluated by running the sample on the FemtoPulse system. For this sample, final post-shearing DNA measurements included a Qubit concentration of 15.8 ng/μL, a yield of 869.00 ng, and an average fragment size of 14.4 kb. The 260/280 spectrophotometric ratio was 2, and the 260/230 ratio was 2.54. The Genomic Quality Number (GQN) was 7.2.

RNA was extracted from leaf tissue of dhAlnCord1 in the Tree of Life Laboratory at the WSI using the RNA Extraction: Automated MagMax™ *mir*Vana protocol. The RNA concentration was assessed using a NanoDrop spectrophotometer and a Qubit Fluorometer using the Qubit RNA Broad-Range Assay kit. Analysis of the integrity of the RNA was done using the Agilent RNA 6000 Pico Kit and Eukaryotic Total RNA assay.

### PacBio HiFi library preparation and sequencing

Library preparation and sequencing were performed at the WSI Scientific Operations core. Library DTOL14377264 was prepared using the SMRTbell Prep Kit 3.0 (Pacific Biosciences) according to the manufacturer’s instructions. The kit includes reagents for end repair/A-tailing, adapter ligation, post-ligation SMRTbell bead clean-up, and nuclease treatment. Size selection and clean-up were performed using diluted AMPure PB beads (Pacific Biosciences). DNA concentration was quantified using a Qubit Fluorometer v4.0 (Thermo Fisher Scientific) and the Qubit 1X dsDNA HS assay kit. Final library fragment size was assessed with the Agilent Femto Pulse Automated Pulsed Field CE Instrument (Agilent Technologies) using the gDNA 55 kb BAC analysis kit. Library DTOL14377264 was sequenced using the Sequel IIe system (Pacific Biosciences, California, USA). The concentration of the library loaded onto the Sequel system was in the range 40–135 pM. SMRT Link software, a PacBio web-based workflow manager, was used to set up and monitor the run, and to perform primary and secondary analysis of the data upon completion.

### Hi-C

***Sample preparation and crosslinking***

Hi-C data were generated from 68 mg of the dhAlnCord1 leaf sample using the Arima-HiC v2 kit (Arima Genomics). Tissue was finely ground using the Covaris cryoPREP Dry Pulverizer (Covaris), and then subjected to nuclei isolation. Nuclei were isolated using a modified protocol based on the Qiagen QProteome Cell Compartment Kit (Qiagen), in which only the Lysis and CE2 buffers were used, with QIAshredder spin columns. After isolation, nuclei were fixed using formaldehyde to a final concentration of 2% to crosslink the DNA. The crosslinked DNA was then digested and biotinylated according to the manufacturer’s instructions. A clean-up step was performed with SPRIselect beads before library preparation. DNA concentration was quantified using the Qubit Fluorometer v4.0 (Thermo Fisher Scientific) and the Qubit HS Assay Kit, following the manufacturer’s instructions.

***Hi-C library preparation and sequencing***

Biotinylated DNA constructs were fragmented using a Covaris E220 sonicator and size selected to 400–600 bp using SPRISelect beads. DNA was enriched with Arima-HiC v2 kit Enrichment beads. End repair, A-tailing, and adapter ligation were carried out with the NEBNext Ultra II DNA Library Prep Kit (New England Biolabs), following a modified protocol where library preparation occurs while DNA remains bound to the Enrichment beads. Library amplification was performed using KAPA HiFi HotStart mix and a custom Unique Dual Index (UDI) barcode set (Integrated DNA Technologies). Depending on sample concentration and biotinylation percentage determined at the crosslinking stage, libraries were amplified with 10–16 PCR cycles. Post-PCR clean-up was performed with SPRISelect beads. Libraries were quantified using the AccuClear Ultra High Sensitivity dsDNA Standards Assay Kit (Biotium) and a FLUOstar Omega plate reader (BMG Labtech).

Prior to sequencing, libraries were normalised to 10 ng/μL. Normalised libraries were quantified again to create equimolar and/or weighted 2.8 nM pools. Pool concentrations were checked using the Agilent 4200 TapeStation (Agilent) with High Sensitivity D500 reagents before sequencing. Sequencing was performed using paired-end 150 bp reads on the Illumina NovaSeq X.

### RNA library preparation and sequencing

Libraries were prepared using the NEBNext® Ultra II Directional RNA Library Prep Kit for Illumina (New England Biolabs), following the manufacturer’s instructions. Poly(A) mRNA was isolated from 100 ng of total RNA using oligo (dT) beads, converted to cDNA, and uniquely indexed; 14 PCR cycles were performed. Libraries were prepared with an intended average fragment size of 100–300 bp. Libraries were quantified, normalised, and pooled equimolarly to a final concentration of 2.8 nM before dilution to 150 pM for loading. Sequencing was carried out on the Illumina NovaSeq X, generating paired-end reads.

### Genome assembly

Prior to assembly of the PacBio HiFi reads, a database of *k*-mer counts ( *k* = 31) was generated from the filtered reads using FastK. GenomeScope2 ( Ranallo-Benavidez *et al.*, 2020) was used to analyse the *k*-mer frequency distributions, providing estimates of genome size, heterozygosity, and repeat content.

The HiFi reads were assembled using Hifiasm in Hi-C phasing mode ( Cheng *et al.*, 2021, 2022), producing two haplotypes. Hi-C reads ( Rao *et al.*, 2014) were mapped to the primary contigs using bwa-mem2 ( Vasimuddin *et al.*, 2019). Contigs were further scaffolded with Hi-C data in YaHS ( Zhou *et al.*, 2023), using the --break option for handling potential misassemblies. The scaffolded assemblies were evaluated using Gfastats ( Formenti *et al.*, 2022), BUSCO ( Manni *et al.*, 2021) and MerquryFK ( Rhie *et al.*, 2020). The organelle genomes were assembled using OATK ( Zhou *et al.*, 2025). The comparator for the plastid assembly was the chloroplast genome of *Alnus cordata* (NC_036751.1).

### Assembly curation

The assembly was decontaminated using the Assembly Screen for Cobionts and Contaminants ( ASCC) pipeline. TreeVal was used to generate the flat files and maps for use in curation. Manual curation was conducted primarily in PretextView and HiGlass ( Kerpedjiev *et al.*, 2018). Scaffolds were visually inspected and corrected as described by Howe *et al*. (2021). Manual corrections included 39 breaks and 52 joins. This reduced the scaffold count by 8.2%, increased the scaffold N50 by 6.8%, and reduced the total assembly length by 0.5%. The curation process is described at sanger-tol/curation-resources . PretextSnapshot was used to generate a Hi-C contact map of the final assembly.

### Assembly quality assessment

The MerquryFK tool ( Rhie *et al.*, 2020) was run in a Singularity container ( Kurtzer *et al.*, 2017) to evaluate *k*-mer completeness and assembly quality for both haplotypes using the *k*-mer database ( *k* = 31) computed prior to genome assembly. The analysis outputs included assembly QV scores and completeness statistics.

The genome was analysed using the BlobToolKit pipeline, a Nextflow implementation of the earlier Snakemake version ( Challis *et al.*, 2020). PacBio reads were aligned to the assembly using minimap2 ( Li, 2018) and processed with SAMtools ( Danecek *et al.*, 2021) to generate coverage tracks. The pipeline runs BUSCO ( Manni *et al.*, 2021) using lineages identified from the NCBI Taxonomy ( Schoch *et al.*, 2020). For the three basal BUSCO lineages, eukaryota, bacteria and archaea, BUSCO genes are aligned to the UniProt Reference Proteomes database ( Bateman *et al.*, 2023) using DIAMOND BLASTp ( Buchfink *et al.*, 2021). The genome assembly is divided into chunks based on the density of BUSCO genes from the closest taxonomic lineage, and each chunk is aligned to the UniProt Reference Proteomes database using DIAMOND BLASTx. Sequences without DIAMOND BLASTx hits are extracted using seqtk and aligned to the NT database using BLASTn ( Altschul *et al.*, 1990). The outputs are consolidated into a BlobDir for visualisation in BlobToolKit. The BlobToolKit pipeline was developed using nf-core tooling ( Ewels *et al.*, 2020) and MultiQC ( Ewels *et al.*, 2016), with containerisation through Docker ( Merkel, 2014) and Singularity ( Kurtzer *et al.*, 2017).

## Genome sequence report

### Sequence data

The genome of a specimen of *Alnus cordata* was sequenced using Pacific Biosciences single-molecule HiFi long reads, generating 13.43 Gb (gigabases) from 1.17 million reads, which were used to assemble the genome. GenomeScope2.0 analysis using a *p* = 2 model gave a *k*-mer-based genome size estimate of 493.14 Mb, with a heterozygosity of 0.61% and repeat content of 32.90% ( Figure 2). Using flow cytometry, the genome size (1C-value) of the sample was estimated to be 0.64 pg, equivalent to 630.00 Mb. These estimates guided expectations for the assembly. Based on the estimated genome size, the sequencing data provided approximately 26× coverage. Hi-C sequencing produced 84.42 Gb from 279.55 million read pairs, which were used to scaffold the assembly. RNA sequencing data were also generated and are available in public sequence repositories. Table 1 summarises the specimen and sequencing details.

**Figure 2.**
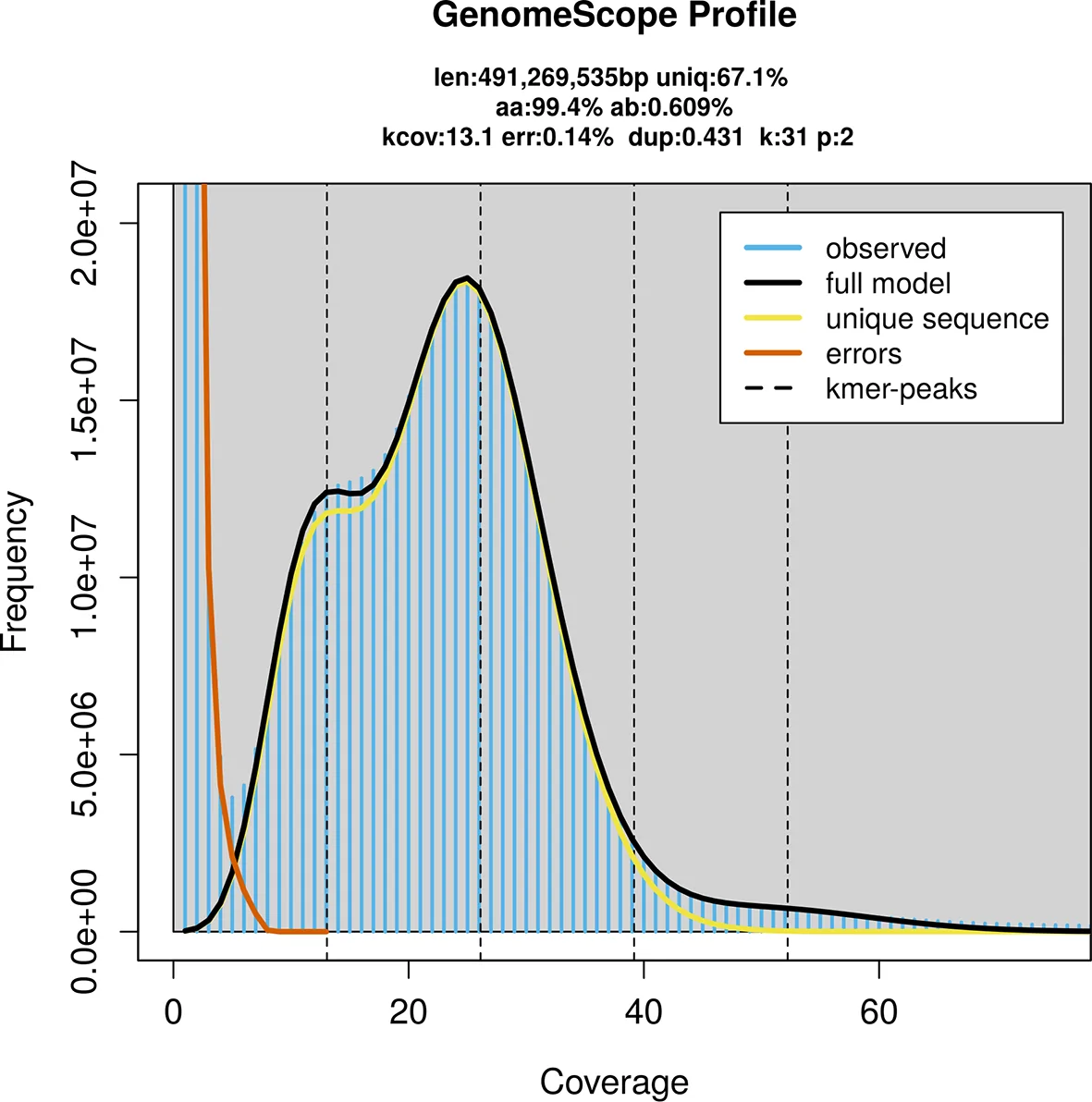
Frequency distribution of *k*-mers generated using GenomeScope2. The plot shows observed and modelled *k*-mer spectra, providing estimates of genome size, heterozygosity, and repeat content based on unassembled sequencing reads.

**Table 1.** Specimen and sequencing data for *Alnus cordata* (BioProject PRJEB73942).

Platform	PacBio HiFi	Hi-C	RNA-seq
**ToLID**	dhAlnCord1	dhAlnCord1	dhAlnCord1
**Specimen ID**	EDTOL05638	EDTOL05638	EDTOL05638
**BioSample (source individual)**	SAMEA114389521	SAMEA114389521	SAMEA114389521
**BioSample (tissue)**	SAMEA114389590	SAMEA114389590	SAMEA114389590
**Tissue**	leaf	leaf	leaf
**Instrument**	Sequel IIe (1 run)	Illumina NovaSeq X	Illumina NovaSeq X
**Run accessions**	ERR12760828	ERR12765201	ERR14792825
**Read count total**	1.17 million reads	279.55 million read pairs	40.07 million read pairs
**Base count total**	13.43 Gb	84.42 Gb	12.10 Gb

### Assembly statistics

The genome was assembled into two haplotypes using Hi-C phasing. Haplotype 1 was curated to chromosome level, while haplotype 2 was assembled to scaffold level. The final assembly has a total length of 468.52 Mb in 142 scaffolds, with 394 gaps, and a scaffold N50 of 33.61 Mb ( Table 2).

**Table 2.** Genome assembly statistics for *Alnus cordata.*

Genome assembly	Haplotype 1	Haplotype 2
**Assembly name**	dhAlnCord1.hap1.2	dhAlnCord1.hap2.2
**Assembly accession**	GCA_965212435.2	GCA_965212605.2
**Assembly level**	chromosome	scaffold
**Span (Mb)**	468.52	468.51
**Number of chromosomes**	14	-
**Number of contigs**	536	482
**Contig N50 (Mb)**	1.92	1.82
**Number of scaffolds**	142	88
**Scaffold N50 (Mb)**	33.61	33.6
**Longest scaffold length (Mb)**	52.2	52.38
**Organelles**	Mitochondrial genomes: 521.58 and 155.53 kb; Plastid genome: 160.76 kb	-

Most of the assembly sequence (98.99%) was assigned to 14 chromosomal-level scaffolds. These chromosome-level scaffolds, confirmed by Hi-C data, are named according to size ( Figure 3; Table 3).

**Figure 3.**
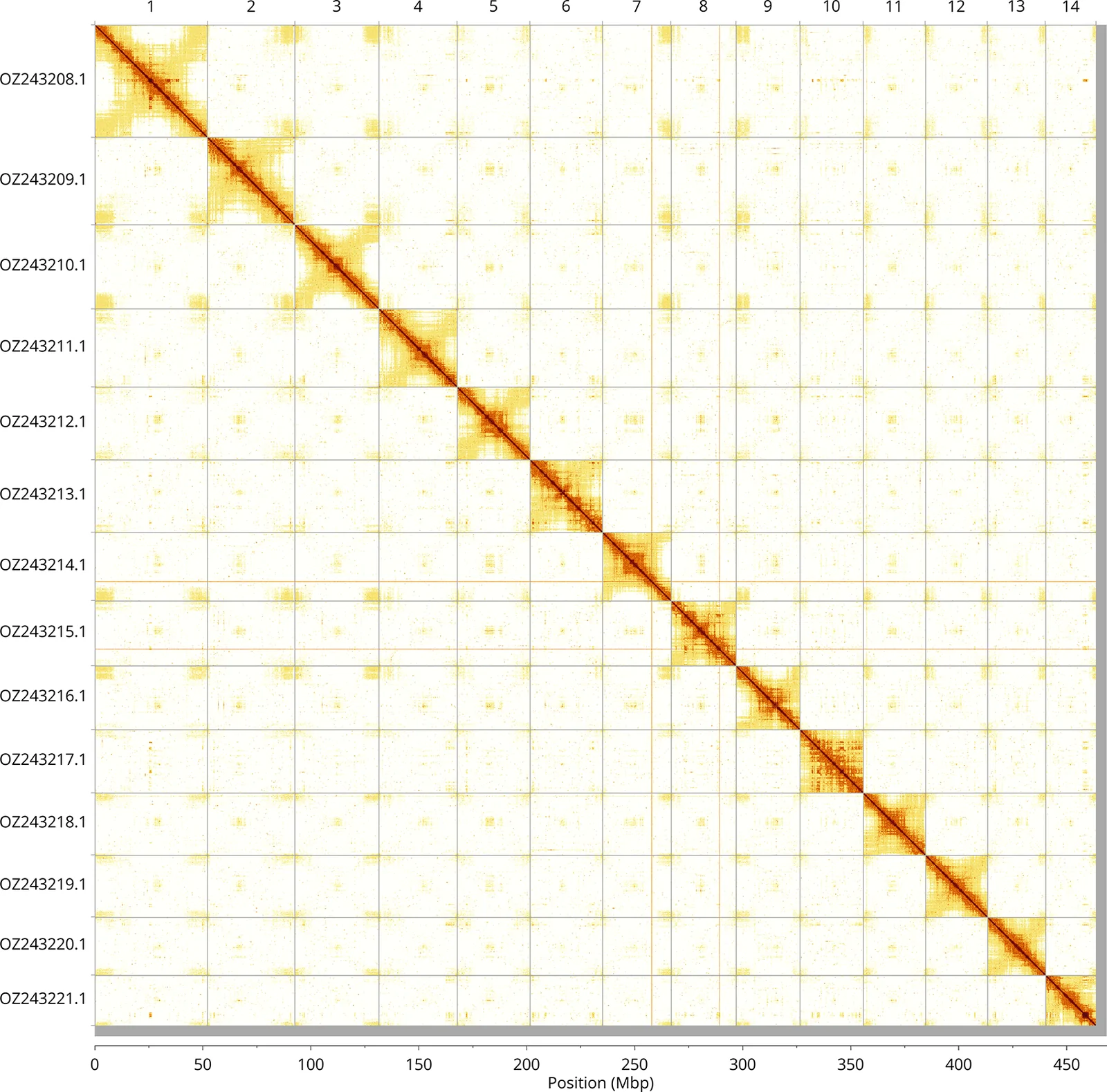
Hi-C contact map of the *Alnus cordata* genome assembly. Assembled chromosomes are shown in order of size and labelled along the axes, with a megabase scale shown below. The plot was generated using PretextSnapshot.

**Table 3.** Chromosomal pseudomolecules in the haplotype 1 genome assembly of *Alnus cordata* dhAlnCord1.

INSDC accession	Molecule	Length (Mb)	GC%
OZ243208.1	1	52.20	36.50
OZ243209.1	2	40.50	37
OZ243210.1	3	38.96	37
OZ243211.1	4	36.21	37
OZ243212.1	5	33.68	37
OZ243213.1	6	33.61	37
OZ243214.1	7	31.74	37
OZ243215.1	8	30.14	36.50
OZ243216.1	9	29.58	37
OZ243217.1	10	29.32	36
OZ243218.1	11	28.78	36.50
OZ243219.1	12	28.77	37
OZ243220.1	13	26.85	37
OZ243221.1	14	23.45	36.50

Two mitochondrial genomes (lengths 521.58 kb [OZ243222.1] and 155.53 kb [OZ243223.1]) and the plastid genome (length 160.76 kb, OZ462988.1) were also assembled. These sequences are included as contigs in the multifasta file of the genome submission and as standalone records.

### Assembly quality metrics

For haplotype 1, the estimated QV is 59.9, and for haplotype 2, 60.3. When the two haplotypes are combined, the assembly achieves an estimated QV of 60.1. The *k*-mer completeness is 87.23% for haplotype 1, 87.42% for haplotype 2, and 98.84% for the combined haplotypes ( Figure 4).

**Figure 4.**
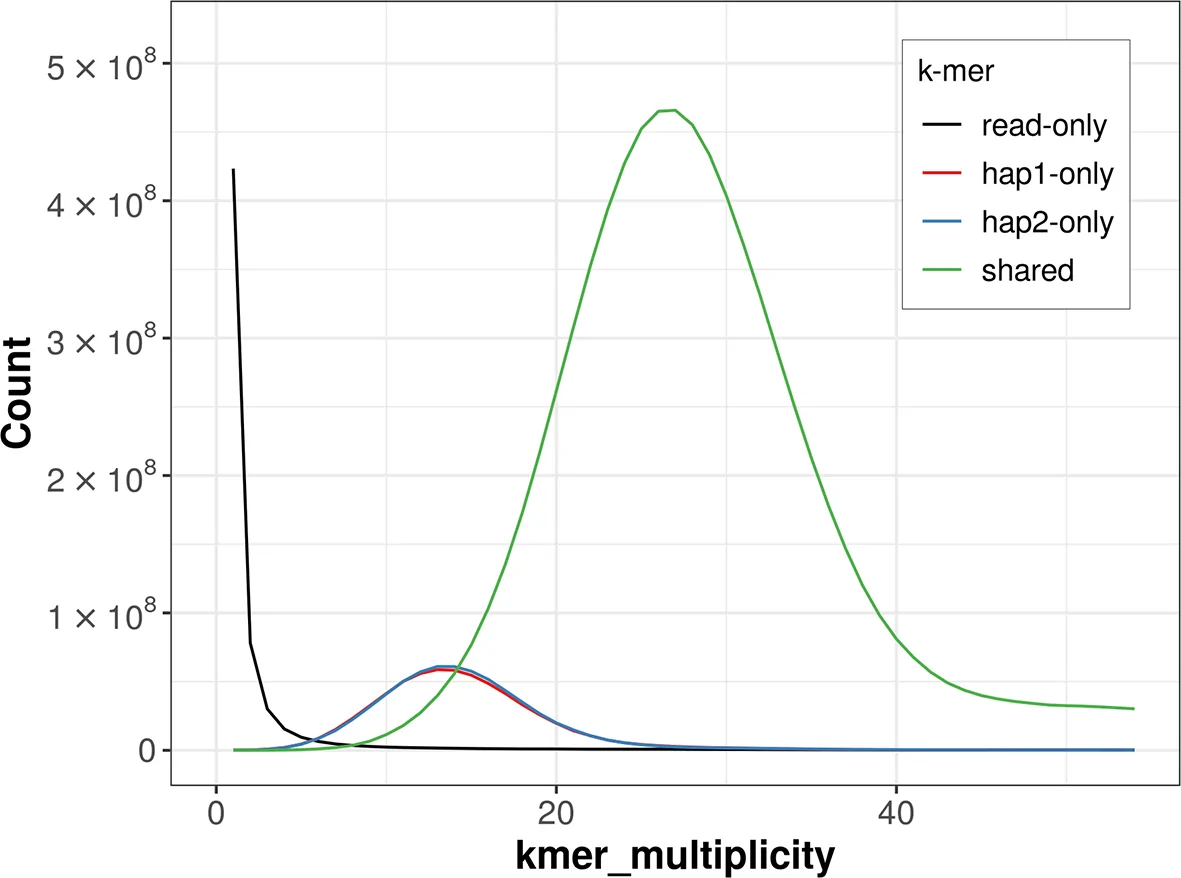
Evaluation of *k*-mer completeness using MerquryFK. This plot illustrates the recovery of *k*-mers from the original read data in the final assemblies. The horizontal axis represents *k*-mer multiplicity, and the vertical axis shows the number of *k*-mers. The black curve represents *k*-mers that appear in the reads but are not assembled. The green curve corresponds to *k*-mers shared by both haplotypes, and the red and blue curves show *k*-mers found only in one of the haplotypes.

BUSCO analysis using the eudicotyledons_odb12 reference set ( *n* = 2,805) identified 99.3% of the expected gene set (single = 96.6%, duplicated = 2.6%) for haplotype 1. The snail plot in Figure 5 summarises the scaffold length distribution and other assembly statistics for haplotype 1. The blob plot in Figure 6 shows the distribution of scaffolds by GC proportion and coverage for haplotype 1.

**Figure 5.**
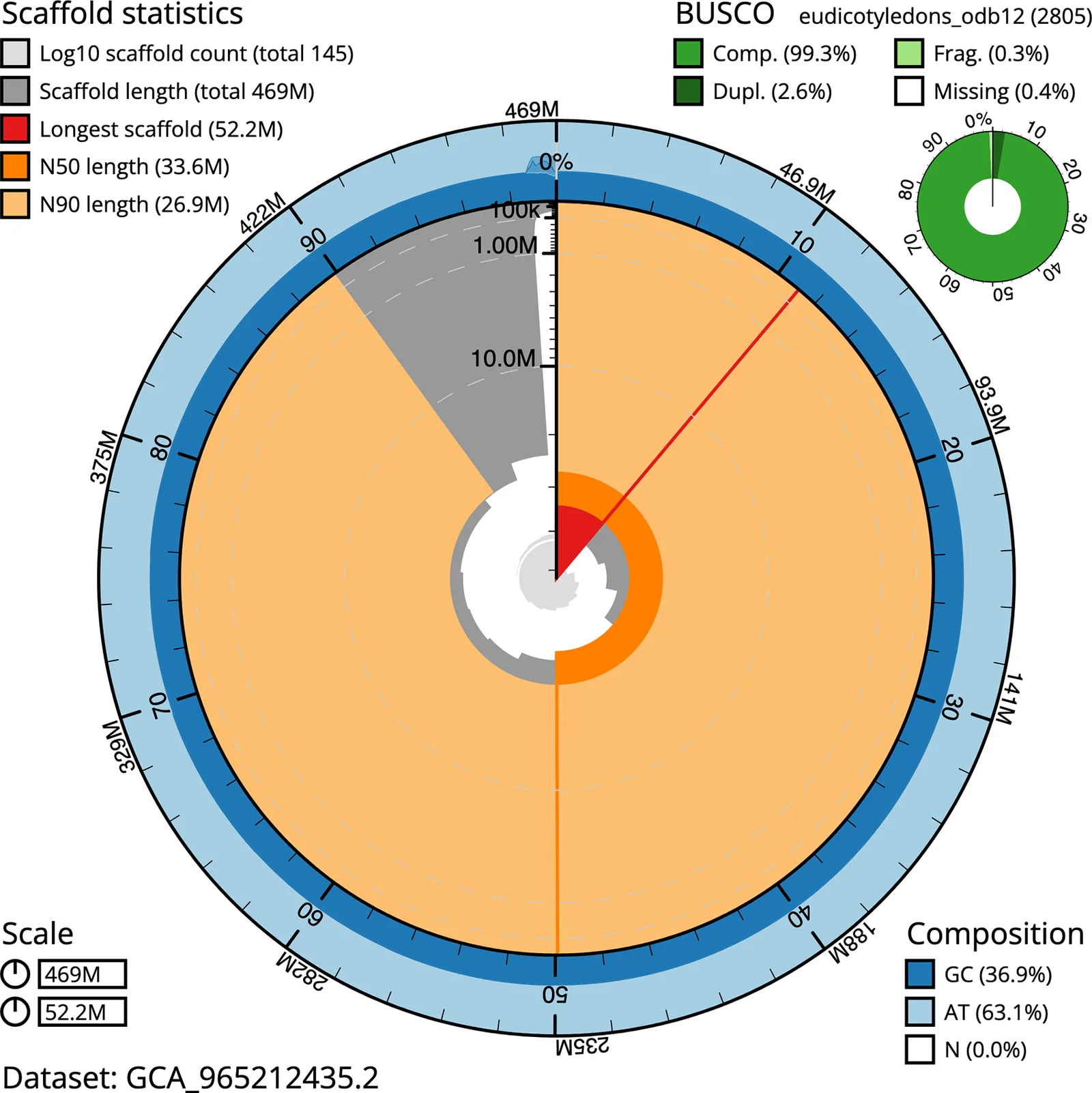
Assembly metrics for dhAlnCord1.hap1.2. The BlobToolKit snail plot provides an overview of assembly metrics and BUSCO gene completeness. The circumference represents the length of the whole genome sequence, and the main plot is divided into 1 000 bins around the circumference. The outermost blue tracks display the distribution of GC, AT, and N percentages across the bins. Scaffolds are arranged clockwise from longest to shortest and are depicted in dark grey. The longest scaffold is indicated by the red arc, and the deeper orange and pale orange arcs represent the N50 and N90 lengths. A light grey spiral at the centre shows the cumulative scaffold count on a logarithmic scale. A summary of complete, fragmented, duplicated, and missing BUSCO genes in the eudicotyledons_odb12 set is presented at the top right. An interactive version of this figure can be accessed on the BlobToolKit viewer.

**Figure 6.**
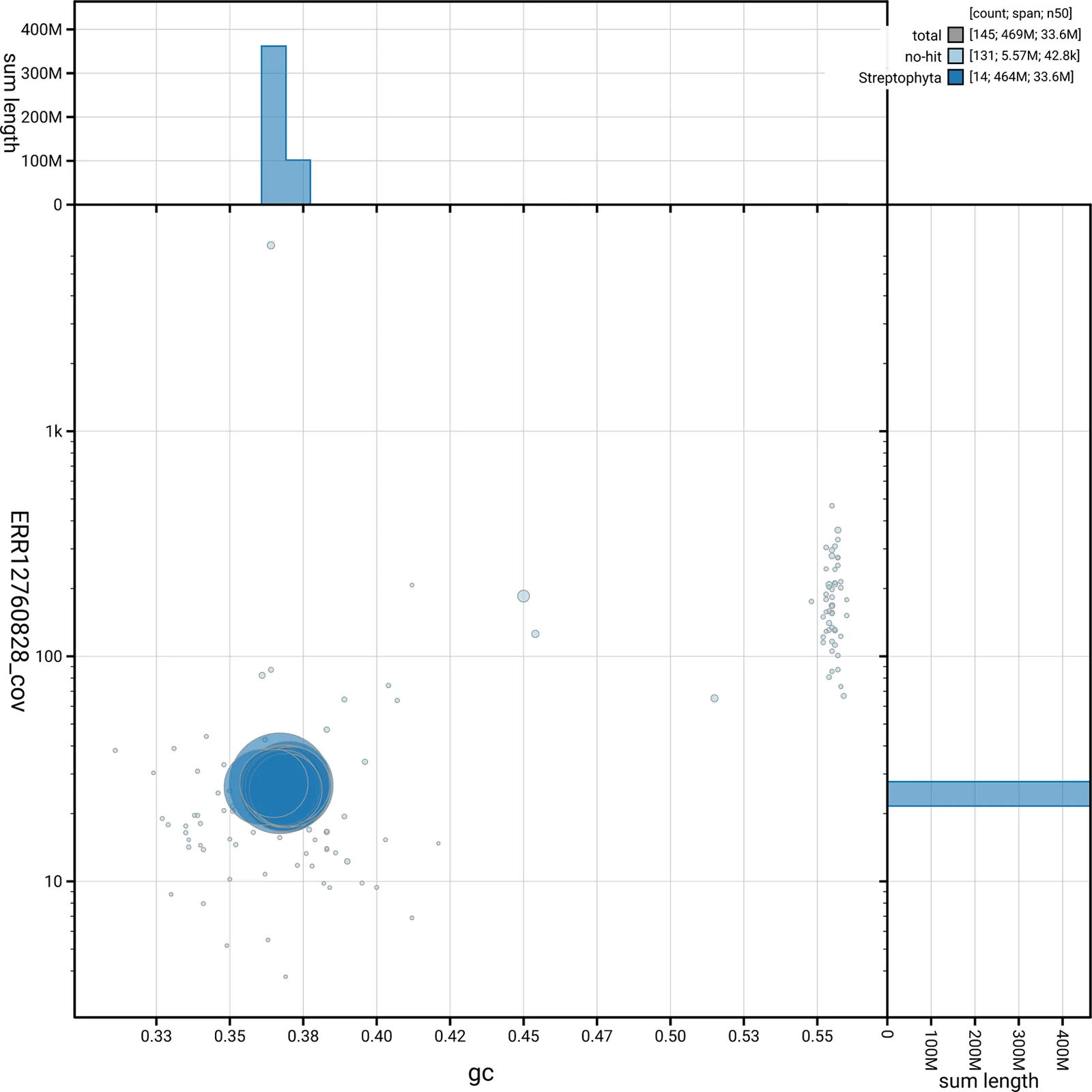
BlobToolKit blob plot for dhAlnCord1.hap1.2. The plot shows base coverage (vertical axis) and GC content (horizontal axis). The circles represent scaffolds, with the size proportional to scaffold length and the colour representing phylum membership. The histograms along the axes display the total length of sequences distributed across different levels of coverage and GC content. An interactive version of this figure is available on the BlobToolKit viewer.

Table 4 lists the assembly metric benchmarks adapted from Rhie *et al.* (2021) and the Earth BioGenome Project (EBP) Report on Assembly Standards. The EBP metric calculated for haplotype 1 is **6.C.Q59**, meeting the recommended 6.C.Q40 reference standard.

**Table 4.** Earth Biogenome Project summary metrics for the *Alnus cordata* assembly.

Measure	Value	Benchmark
EBP summary (haplotype 1)	6.C.Q59	6.C.Q40
Contig N50 length	1.92 Mb	≥ 1 Mb
Scaffold N50 length	33.61 Mb	= chromosome N50
Consensus quality (QV)	Haplotype 1: 59.9; haplotype 2: 60.3; combined: 60.1	≥ 40
*k*-mer completeness	Haplotype 1: 87.23%; Haplotype 2: 87.42%; combined: 98.84%	> 90%
BUSCO	Haplotype 1: C:99.3% [S:96.6%, D:2.6%], F:0.3%, M:0.4%, n:2,805; Haplotype 2: C:99.4% [S:97.1%, D:2.2%], F:0.3%, M:0.3%, n:2,805	S > 90%; D < 5%
Percentage of assembly assigned to chromosomes	98.99%	≥ 90%

*Note*: The EBP summary uses log10(Contig N50); chromosome-level (C) or log10(Scaffold N50); Q (Merqury QV). BUSCO: C = complete; S = single-copy; D = duplicated; F = fragmented; M = missing; n = orthologues.

**Table 5.** Software versions and sources used for *Alnus cordata.*

Software	Version	Source
BLAST	2.14.0	https://ftp.ncbi.nlm.nih.gov/blast/executables/blast+/
BlobToolKit	4.4.5	https://github.com/genomehubs/blobtoolkit
blobtk	0.5.1	https://github.com/genomehubs/blobtk
BUSCO	6.0.0; 5.7.1	https://gitlab.com/ezlab/busco
bwa-mem2	2.2.1	https://github.com/bwa-mem2/bwa-mem2
DIAMOND	2.1.8	https://github.com/bbuchfink/diamond
fasta_windows	0.2.4	https://github.com/tolkit/fasta_windows
FastK	1.1	https://github.com/thegenemyers/FASTK
GenomeScope2.0	2.0.1	https://github.com/tbenavi1/genomescope2.0
Gfastats	1.3.6	https://github.com/vgl-hub/gfastats
Hifiasm	0.19.8-r603	https://github.com/chhylp123/hifiasm
HiGlass	1.13.4	https://github.com/higlass/higlass
MerquryFK	1.1.0-c1	https://github.com/thegenemyers/MERQURY.FK
Minimap2	2.28-r1209	https://github.com/lh3/minimap2
Oatk	1.0	https://github.com/c-zhou/oatk
MultiQC	1.14; 1.17 and 1.18	https://github.com/MultiQC/MultiQC
Nextflow	23.10.0; 24.10.4	https://github.com/nextflow-io/nextflow
PretextSnapshot	0.0.6	https://github.com/sanger-tol/PretextSnapshot
PretextView	1.0.3	https://github.com/sanger-tol/PretextView
samtools	1.17; 1.2; 1.20; 1.21	https://github.com/samtools/samtools
sanger-tol/ascc	0.1.0	https://github.com/sanger-tol/ascc
sanger-tol/blobtoolkit	v0.10.2	https://github.com/sanger-tol/blobtoolkit
sanger-tol/curationpretext	1.4.2	https://github.com/sanger-tol/curationpretext
Seqtk	1.4-r122	https://github.com/lh3/seqtk
Singularity	3.9.0	https://github.com/sylabs/singularity
TreeVal	1.2.1	https://github.com/sanger-tol/treeval
YaHS	1.2a.2	https://github.com/c-zhou/yahs

## Author information

• Members of the Royal Botanic Garden Edinburgh Genome Acquisition Lab
• Members of the Plant Genome Sizing Collective
• Members of the Darwin Tree of Life Barcoding collective
• Members of the Wellcome Sanger Institute Tree of Life Management, Samples and Laboratory team
• Members of Wellcome Sanger Institute Scientific Operations – Sequencing Operations
• Members of the Wellcome Sanger Institute Tree of Life Core Informatics team
• Members of the Tree of Life Core Informatics collective
• Members of the Darwin Tree of Life Consortium

## Wellcome Sanger Institute – Legal and Governance

The materials that have contributed to this genome note have been supplied by a Darwin Tree of Life Partner. The submission of materials by a Darwin Tree of Life Partner is subject to the **‘Darwin Tree of Life Project Sampling Code of Practice’**, which can be found in full on the Darwin Tree of Life website. By agreeing with and signing up to the Sampling Code of Practice, the Darwin Tree of Life Partner agrees they will meet the legal and ethical requirements and standards set out within this document in respect of all samples acquired for, and supplied to, the Darwin Tree of Life Project. Further, the Wellcome Sanger Institute employs a process whereby due diligence is carried out proportionate to the nature of the materials themselves, and the circumstances under which they have been/are to be collected and provided for use. The purpose of this is to address and mitigate any potential legal and/or ethical implications of receipt and use of the materials as part of the research project, and to ensure that in doing so we align with best practice wherever possible. The overarching areas of consideration are:

• Ethical review of provenance and sourcing of the material
• Legality of collection, transfer and use (national and international)

Each transfer of samples is further undertaken according to a Research Collaboration Agreement or Material Transfer Agreement entered into by the Darwin Tree of Life Partner, Genome Research Limited (operating as the Wellcome Sanger Institute), and in some circumstances, other Darwin Tree of Life collaborators.

## Data Availability

European Nucleotide Archive: Alnus cordata. Accession number PRJEB73942; https://identifiers.org/ena.embl/PRJEB73942. The genome sequence is released openly for reuse. The *Alnus cordata* genome sequencing initiative is part of the Darwin Tree of Life Project ( PRJEB40665) and the Sanger Institute Tree of Life Programme ( PRJEB43745). All raw sequence data and the assembly have been deposited in INSDC databases. The genome will be annotated using available RNA-Seq data and presented through the Ensembl pipeline at the European Bioinformatics Institute. Raw data and assembly accession identifiers are reported in Tables 1 and 2. Pipelines used for genome assembly at the WSI Tree of Life are available at https://pipelines.tol.sanger.ac.uk/pipelines. Table 5 lists software versions used in this study.

## References

[ref1] Altschul SF Gish W Miller W : Basic Local Alignment Search Tool. *J. Mol. Biol.* 1990;215(3):403–410. 2231712 10.1016/S0022-2836(05)80360-2

[ref2] Bateman A Martin M-J Orchard S : UniProt: The Universal Protein Knowledgebase in 2023. *Nucleic Acids Res.* 2023;51(D1):D523–D531. 36408920 10.1093/nar/gkac1052 PMC9825514

[ref3] Brasier CM Kirk SA Delcan J : *Phytophthora alni* sp. Nov. And its variants: Designation of emerging heteroploid hybrid pathogens spreading on *Alnus* trees. *Mycol. Res.* 2004;108(10):1172–84. 10.1017/S0953756204001005 15535068

[ref4] Buchfink B Reuter K Drost H-G : Sensitive protein alignments at tree-of-life scale using DIAMOND. *Nat. Methods.* 2021;18(4):366–368. 33828273 10.1038/s41592-021-01101-x PMC8026399

[ref5] Caudullo G Mauri A : *Alnus cordata* in Europe: Distribution, habitat, usage and threats. San-Miguel-Ayanz J , editor. *European Atlas of Forest Tree Species.* Luxembourg: Publications Office of the European Union;2016;e015443+. Reference Source

[ref6] Challis R Richards E Rajan J : BlobToolKit – interactive quality assessment of genome assemblies. *G3: Genes, Genomes, Genetics.* 2020;10(4):1361–1374. 32071071 10.1534/g3.119.400908 PMC7144090

[ref7] Chase MW Hills HH : Silica gel: An ideal material for field preservation of leaf samples for DNA studies. *Taxon.* 1991;40(2):215–220. 10.2307/1222975

[ref8] Cheng H Concepcion GT Feng X : Haplotype-resolved *de novo* assembly using phased assembly graphs with Hifiasm. *Nat. Methods.* 2021;18(2):170–175. 33526886 10.1038/s41592-020-01056-5 PMC7961889

[ref9] Cheng H Jarvis ED Fedrigo O : Haplotype-resolved assembly of diploid genomes without parental data. *Nat. Biotechnol.* 2022;40(9):1332–1335. 35332338 10.1038/s41587-022-01261-x PMC9464699

[ref10] Danecek P Bonfield JK Liddle J : Twelve years of SAMtools and BCFtools. *GigaScience.* 2021;10(2):giab008. 33590861 10.1093/gigascience/giab008 PMC7931819

[ref11] Darwin Tree of Life Project Consortium: Sequence locally, think globally: The Darwin Tree of Life Project. *Proc. Natl. Acad. Sci. USA.* 2022;119(4):e2115642118. 35042805 10.1073/pnas.2115642118 PMC8797607

[ref12] Ducci F Tani A : *EUFORGEN Technical Guidelines for Genetic Conservation and Use of Italian Alder (Alnus Cordata).* Rome, Italy: Bioversity International;2009. Reference Source

[ref13] Ewels P Magnusson M Lundin S : MultiQC: Summarize analysis results for multiple tools and samples in a single report. *Bioinformatics.* 2016;32(19):3047–3048. 27312411 10.1093/bioinformatics/btw354 PMC5039924

[ref14] Ewels PA Peltzer A Fillinger S : The nf-core framework for community-curated bioinformatics pipelines. *Nat. Biotechnol.* 2020;38(3):276–278. 32055031 10.1038/s41587-020-0439-x

[ref15] Formenti G Abueg L Brajuka A : Gfastats: Conversion, evaluation and manipulation of genome sequences using assembly graphs. *Bioinformatics.* 2022;38(17):4214–4216. 35799367 10.1093/bioinformatics/btac460 PMC9438950

[ref16] Howard C Denton A Jackson B : On the path to reference genomes for all biodiversity: Lessons learned and laboratory protocols created in the Sanger Tree of Life core laboratory over the first 2000 species. *bioRxiv.* 2025. 10.1101/2025.04.11.648334 PMC12548527 41129326

[ref17] Howe K Chow W Collins J : Significantly improving the quality of genome assemblies through curation. *GigaScience.* 2021;10(1):giaa153. 33420778 10.1093/gigascience/giaa153 PMC7794651

[ref18] Kerpedjiev P Abdennur N Lekschas F : HiGlass: Web-based visual exploration and analysis of genome interaction maps. *Genome Biol.* 2018;19(1):125. 30143029 10.1186/s13059-018-1486-1 PMC6109259

[ref19] Kurtzer GM Sochat V Bauer MW : Singularity: Scientific containers for mobility of compute. *PLOS ONE.* 2017;12(5):e0177459. 28494014 10.1371/journal.pone.0177459 PMC5426675

[ref20] Li H : Minimap2: Pairwise alignment for nucleotide sequences. *Bioinformatics.* 2018;34(18):3094–3100. 29750242 10.1093/bioinformatics/bty191 PMC6137996

[ref21] Loureiro J Rodriguez E Doležel J : Two new nuclear isolation buffers for plant DNA flow cytometry: A test with 37 species. *Ann. Bot.* 2007;100(4):875–888. 17684025 10.1093/aob/mcm152 PMC2749623

[ref22] Manni M Berkeley MR Seppey M : BUSCO update: Novel and streamlined workflows along with broader and deeper phylogenetic coverage for scoring of eukaryotic, prokaryotic, and viral genomes. *Mol. Biol. Evol.* 2021;38(10):4647–4654. 34320186 10.1093/molbev/msab199 PMC8476166

[ref23] Merkel D : Docker: Lightweight Linux containers for consistent development and deployment. *Linux J.* 2014;2014(239):2. 10.5555/2600239.2600241

[ref24] Obermayer R Leitch IJ Hanson L : Nuclear DNA C-values in 30 species double the familial representation in pteridophytes. *Ann. Bot.* 2002;90(2):209–217. 12197518 10.1093/aob/mcf167 PMC4240412

[ref25] Pellicer J Powell RF Leitch IJ : The application of flow cytometry for estimating genome size, ploidy level endopolyploidy, and reproductive modes in plants. Besse P , editor. *Methods in Molecular Biology.* New York, NY:2021; Vol.2222:325–61. 33301101 10.1007/978-1-0716-0997-2_17

[ref26] Pollegioni P Marchesini A Gaudet M : What shapes the genetic diversity of the *Alnus cordata* species across its Italian native range? Informing conservation strategies. *Ecol. Evol.* 2025;15(9):e72018. 40896094 10.1002/ece3.72018 PMC12399267

[ref27] Ranallo-Benavidez TR Jaron KS Schatz MC : GenomeScope 2.0 and Smudgeplot for reference-free profiling of polyploid genomes. *Nat. Commun.* 2020;11(1):1432. 32188846 10.1038/s41467-020-14998-3 PMC7080791

[ref28] Rao SSP Huntley MH Durand NC : A 3D map of the human genome at kilobase resolution reveals principles of chromatin looping. *Cell.* 2014;159(7):1665–1680. 25497547 10.1016/j.cell.2014.11.021 PMC5635824

[ref29] Ratnasingham S Hebert PDN : BOLD: The Barcode of Life Data System (http://www.barcodinglife.org). *Mol. Ecol. Notes.* 2007;7(3):355–364. 18784790 10.1111/j.1471-8286.2007.01678.x PMC1890991

[ref30] Rhie A McCarthy SA Fedrigo O : Towards complete and error-free genome assemblies of all vertebrate species. *Nature.* 2021;592(7856):737–746. 33911273 10.1038/s41586-021-03451-0 PMC8081667

[ref31] Rhie A Walenz BP Koren S : Merqury: Reference-free quality, completeness, and phasing assessment for genome assemblies. *Genome Biol.* 2020;21(1):245. 32928274 10.1186/s13059-020-02134-9 PMC7488777

[ref32] Rice A Mayrose I : The Chromosome Counts Database (CCDB). *Plant Genomic and Cytogenetic Databases.* New York, NY: Humana;2023; Vol.2703:123–29. 37646942 10.1007/978-1-0716-3389-2_10

[ref33] Schoch CL Ciufo S Domrachev M : NCBI taxonomy: A comprehensive update on curation, resources and tools. *Database.* 2020;2020:baaa062. 32761142 10.1093/database/baaa062 PMC7408187

[ref34] Smeriglio A D’Angelo V Denaro M : Polyphenol characterization, antioxidant and skin whitening properties of *Alnus cordata* stem bark. *Chem. Biodivers.* 2019;16(9):e1900314. 31397975 10.1002/cbdv.201900314

[ref35] Twyford AD Beasley J Barnes I : A DNA barcoding framework for taxonomic verification in the Darwin Tree of Life Project. *Wellcome Open Research.* 2024;9:339. 39386966 10.12688/wellcomeopenres.21143.1 PMC11462125

[ref36] Vasimuddin M Misra S Li H : Efficient architecture-aware acceleration of BWA-MEM for multicore systems. *2019 IEEE International Parallel and Distributed Processing Symposium (IPDPS).* IEEE;2019;314–324. 10.1109/IPDPS.2019.00041

[ref37] Villani F Castellana S Beritognolo I : Genetic variability of *Alnus cordata* (Loisel.) Duby populations and introgressive hybridization with *A. glutinosa* (L.) Gaertn. In Southern Italy: Implication for conservation and management of genetic resources. *Forests.* 2021;12(6):655. 10.3390/f12060655

[ref38] Vít P Douda J Krak K : Two new polyploid species closely related to *Alnus glutinosa* in Europe and North Africa – an analysis based on morphometry, karyology, flow cytometry and microsatellites. *Taxon.* 2017;66(3):567–583. 10.12705/663.4

[ref39] Zhou C Brown M Blaxter M : Oatk: A de novo assembly tool for complex plant organelle genomes. *Genome Biol.* 2025;26(1):235. 40775726 10.1186/s13059-025-03676-6 PMC12329965

[ref40] Zhou C McCarthy SA Durbin R : YaHS: Yet another Hi-C scaffolding tool. *Bioinformatics.* 2023;39(1):btac808. 36525368 10.1093/bioinformatics/btac808 PMC9848053

