# Supplementary material for: The genome sequence of Alnus cordata (Loisel. Duby, 1828) (Fagales: Betulaceae)
Source: Wellcome Open Res. 2026 Sep 16;11:680. [Version 1] doi: 10.12688/wellcomeopenres.27529.1 (PMC13684016; doi:10.12688/wellcomeopenres.27529.1)
Supplement: Peer review history

# Reviewer report: Reviewer response for version 1

**DOI:** 10.21956/wellcomeopenres.30300.r171584

**Published:** 2026-10-20

**Authors:** Nils Stein

**Affiliations:** 1 Leibniz Institute of Plant Genetics and Crop Plant Research (IPK), Seeland, Germany

Ruhsam et al provide an assembly for Alnus cordata as part of the DToL project. They use the established and well documented data generation, sequencing and annotation pipeline as well as data standards that are routinely applied to sequencing projects within DToL.

 The sequenced specimen is obviously carrying a heterozygous diploid genome, hence it is important to properly refer to haploid/diploid genome when reporting genome size and sequencing coverage. The currently estimated genome sequencing coverage of 26x Hifi is referring to haploid genome size. For assembly and phasing of a heterozygous genome it is recommended to use 20-fold diploid coverage.

 The presented data still provides a high quality reference dataset for the species, however, with a limited value for comparative phylogenomic analyses.

Are sufficient details of methods and materials provided to allow replication by others?

Yes

Is the rationale for creating the dataset(s) clearly described?

Yes

Are the datasets clearly presented in a useable and accessible format?

Yes

Are the protocols appropriate and is the work technically sound?

Yes

Reviewer Expertise:

plant biology, genetics, genomics

I confirm that I have read this submission and believe that I have an appropriate level of expertise to confirm that it is of an acceptable scientific standard.

# Reviewer report: Reviewer response for version 1

**DOI:** 10.21956/wellcomeopenres.30300.r168920

**Published:** 2026-10-15

**Authors:** Aureliano Bombarely

**Affiliations:** 1 Institute for Molecular and Cellular Plant Biology (IBMCP, CSIC-UPV), Valencia, Spain

**Summary of the key results**

 The manuscript titled “The genome sequence of *Alnus cordata* (Loisel. Duby, 1828) (Fagales: Betulaceae)” describes the genome sequencing, and assembly of the species tree, *Alnus cordata*. The estimated genome size was 0.63 Gb (by flow cytometry) and 0.49 Gb with a heterozygosity of 0.61% (by K-mer profiling). The assembly produced two haplotypes with similar assembly sizes of 0.47 Gb. Almost 99% of the assembly was scaffolded into 14 chromosomes. Combined Kmer completeness was 98.84% with a BUSCO score of 99.3%. The assembly was categorized as 6.C.Q59 according the EBP standards. No genome annotation was produced.

**Overall evaluation**

 The assembly metrics looks fine. The methodology has been described in detail. There is not any concern about the presentation of the data. The HiC scaffolding looks good; no uncovered regions or miss-assemblies can be appreciated by a visual inspection. The contamination screening does not show obvious contaminants. The QC metrics are good supporting the tagging of 6.C.Q59 according the EBP standards. The assemblies are available at NCBI.

**Discretionary changes**

- **Report numbers of gaps per chromosome**: Table 3 could incorporate information about the number of gaps per chromosome to give a better idea about the incompleteness of this chromosome-level assembly.
- **A telomere analysis on the two haplotypes revealed only six telomeres missing. **hap1 OZ243209 (3’), hap1 OZ243215 (3’), hap1 OZ243220 5’, hap2 CBDBVM007 (3’), hap2 CBDBVM008 (both ends), and hap2 CBDBVM010 (5’). This, rather than a problem is a good indication of completeness - hap1: 25/28 ends (89.3%), - hap2: 24/28 ends (85.7%). 

Are sufficient details of methods and materials provided to allow replication by others?

Yes

Is the rationale for creating the dataset(s) clearly described?

Yes

Are the datasets clearly presented in a useable and accessible format?

Yes

Are the protocols appropriate and is the work technically sound?

Yes

Reviewer Expertise:

Plant Genome Evolution, Genome Annotation

I confirm that I have read this submission and believe that I have an appropriate level of expertise to confirm that it is of an acceptable scientific standard.
